# Pulmonary arteriovenous malformations presenting as difficult-to-control asthma: a case report

**DOI:** 10.1186/1752-1947-7-32

**Published:** 2013-01-25

**Authors:** Marta Navratil, Vinko Vidjak, Filip Rubić, Damir Erceg, Mirjana Turkalj

**Affiliations:** 1 Reference Center for Clinical Allergology of the Ministry of Health and Social Welfare, Srebrnjak Children’s Hospital, Srebrnjak 100, HR-10000, Zagreb, Croatia; 2 Clinical Department of Diagnostic and Interventional Radiology, Clinical Hospital Merkur, Zajčeva 19, HR-10000, Zagreb, Croatia

**Keywords:** Asthma, Dyspnea, Hypoxemia, Pulmonary arteriovenous malformation

## Abstract

**Introduction:**

Although pulmonary arteriovenous malformations are relatively rare disorders, they are an important part of the differential diagnosis of common pulmonary problems, such as hypoxemia, dyspnea on exertion and pulmonary nodules.

**Case presentation:**

An 11-year-old Croatian boy of Mediterranean origin with a history of asthma since childhood was admitted to our hospital for evaluation of difficult-to-control asthma during the previous six months. A chest X-ray showed a homogeneous soft tissue mass in the lingual area. Computed tomography angiography of the thorax showed two pulmonary arteriovenous malformations, one on each side of the lungs. Diagnosis of hereditary hemorrhagic telangiectasia was made clinically by Curaçao criteria. Genetic analysis revealed a mutation in the endoglin gene. The patient was treated with embolotherapy with good clinical outcome.

**Conclusion:**

We present a case of pulmonary arteriovenous malformations masquerading as refractory asthma.

## Introduction

Pulmonary arteriovenous malformations (PAVM[s]) are abnormal communications between the pulmonary artery and vein [1]. Although these lesions are not a common clinical problem, they are an important part of the differential diagnosis of common pulmonary problems such as hypoxemia, dyspnea on exertion and pulmonary nodules.

Asthma is the most common chronic disease in childhood characterized by coughing, wheezing, dyspnea, chest tightness or pressure, and chest pain. The diagnosis of asthma in children requires a careful review of the child's current and past medical history, family history, a physical examination, results of lung function tests (spirometry, bronchodilatation test, bronchial challenge test) and finally a therapeutic trial of medications [2]. Difficult-to-control asthma is a heterogeneous disorder characterized by persistent symptoms of dyspnea, reduced exercise tolerance, and frequent emergency visits despite treatment with high doses of inhaled or oral corticosteroids and long-acting bronchodilators [3].

The aim of this work (study) was to present the case of a patient with PAVM that had been misdiagnosed as false difficult-to-control asthma.

## Case presentation

An 11-year-old Croatian boy of Mediterranean origin with a history of asthma since childhood was admitted to our hospital for evaluation of difficult-to-control asthma during the previous six months. He complained of dyspnea on exertion and several asthma exacerbations, one of which required hospitalization the previous month. He had been treated with tapering doses of systemic corticosteroids for each exacerbation without improvement of chronic hypoxemia and symptoms. His current medications included: fluticasone and salmeterol 250μg/50μg twice daily, montelukast five mg once daily and salbutamol on an as-needed basis, which he currently used four times daily.

General patient history was positive on atopic diseases, as an infant he had atopic dermatitis with cow's milk allergy. He had also suffered from common epistaxis since childhood. At the age of six he was diagnosed with asthma because of a history of recurrent wheezing episodes and positive bronchoprovocation test. A skin prick test revealed sensitivity to house dust mite and grass pollen. Since the diagnosis of asthma had been established the patient was regularly followed-up by a pediatric pulmonologist and during the last couple of years his asthma was adequately controlled. He was symptom free, without prophylaxis and without the need for rescue medication. He complained of a poor appetite and frequent headaches two months prior to hospitalization.

At hospital admission, his general condition was slightly disturbed; he was hypoxic at rest (saturation level of oxygen in hemoglobin (SaO_2_) 92%), without improvement to oxygen supplemental therapy. Furthermore, there were discontinuous systolic murmurs (two out of six) along his left sternum, slightly decreased breath sounds on the right side of his lungs, two telangiectases on his left cheek and discrete telangiectases on his back; skin and visible mucous membranes were pale to cyanotic.

**Figure 1:**
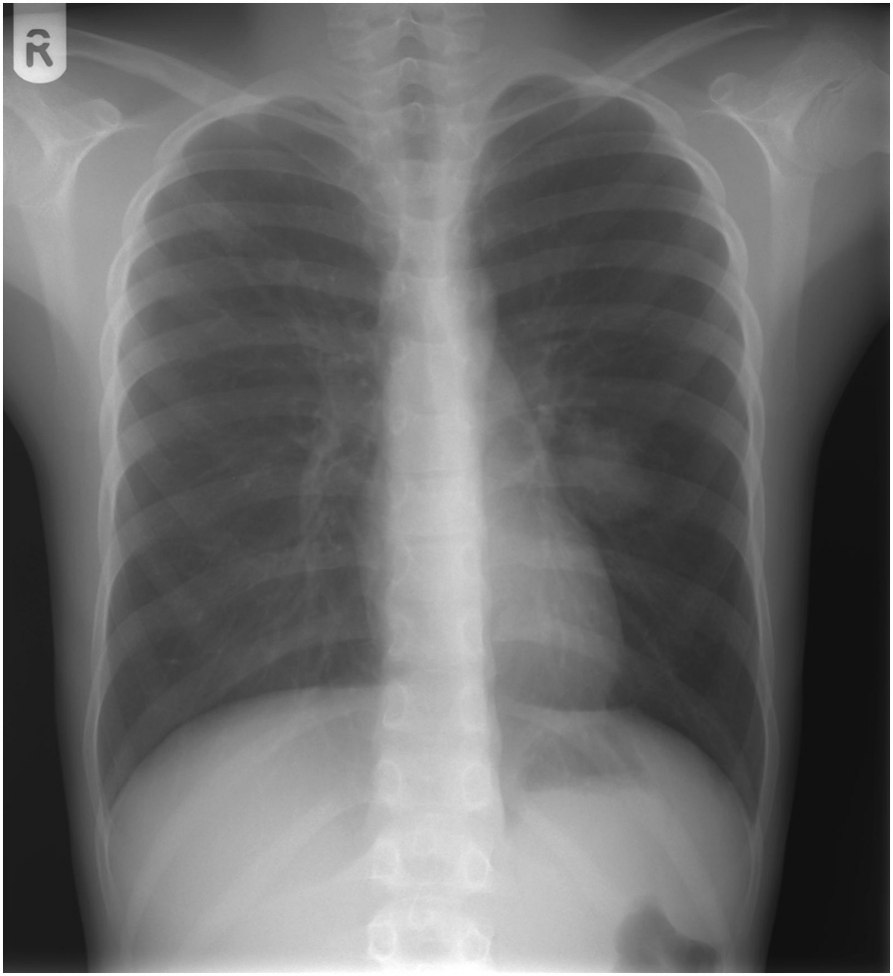
Chest X-ray showed a homogeneous soft tissue mass 20×30mm in the lingual area.

**Figure 2:**
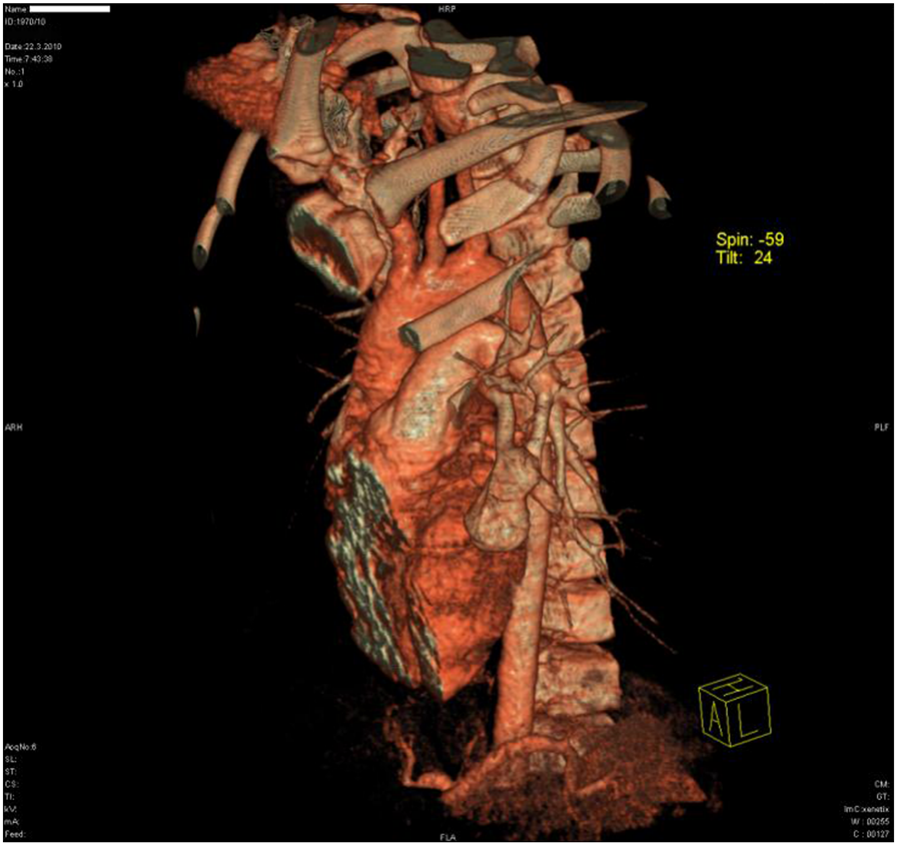
Three-dimensional computed tomography angiography revealed a 35-mm vascular malformation within the perihilar left upper lobe with a single feeding artery originating from the pulmonary artery and a single draining vein.

**Figure 3:**
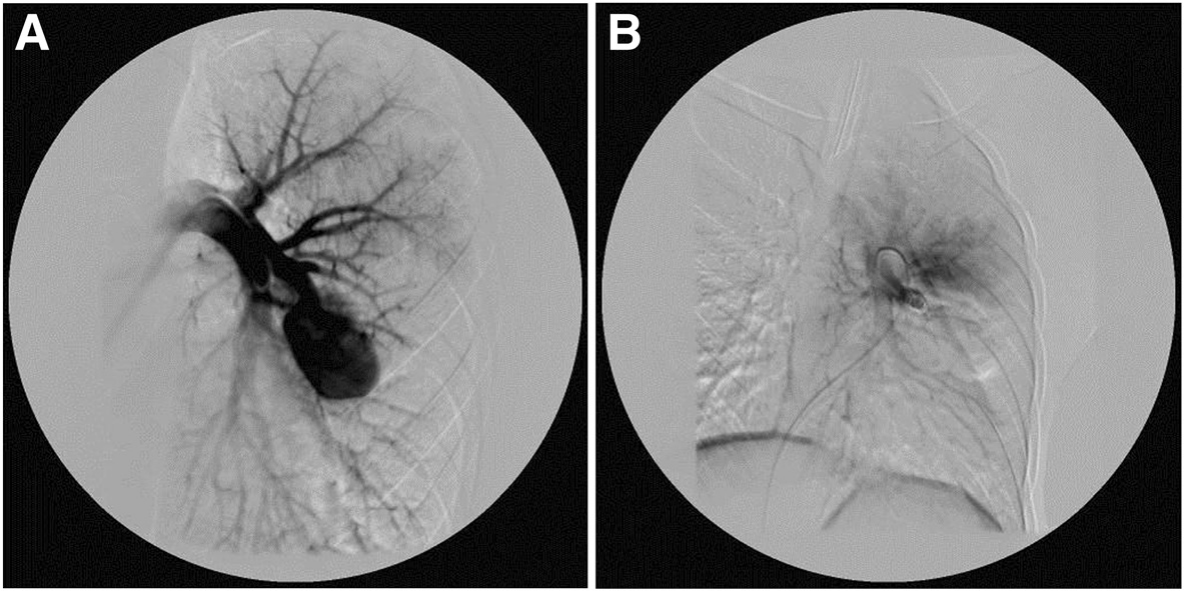
**A. Arteriovenous malformation and aneurism of the left pulmonary artery segmental branch. B**. Status post-embolization of the left pulmonary artery segmental branch with arteriovenous malformation occlusion.

**Figure 4:**
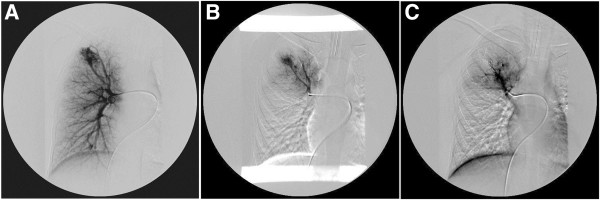
**A. Arteriovenous malformation of the right pulmonary artery segmental branch in the upper lobe with two feeding arteries. B**. Status post one arteriovenous malformation (AVM) feeding artery embolization of the right upper lobe. **C**. Status post second AVM feeding artery embolization in the right upper lobe.

A body plethysmography showed forced vital capacity (FVC) 2.65L (79%), forced expiratory volume in one second (FEV_1_) 2.61L (97%), FEV_1_/FVC 95; airways resistance 0.23L (94%), expiratory airway resistance 0.38L (155%), airway conductance 4.28L (106%), residual volume 1.57L (156%), total lung capacity 4.26L (97%); post-bronchodilator studies FEV_1_ 2.74L (increase of 5%); FVC 2.83L (increase of 5%). The diffusing capacity of his lungs for carbon monoxide (DLCO) was 61%. A bronchoscopy revealed compression of the superior lingular bronchus. A chest X-ray showed a homogeneous soft tissue mass 20×30mm at the left upper lobe of the left lung (Figure 1). Transthoracic echocardiography showed normal cardiac structures with no evidence of pulmonary hypertension. A computed tomography (CT) pulmonary angiogram showed a 35-mm arteriovenous malformation (AVM) within the patient’s perihilar left upper lobe and a small AVM within the apical segment of the right lobe (Figure 2). The left perihilar AVM had a single feeding artery and a huge aneurism. On the right apical segment were two feeding arteries (Figures 3, 4).

Genetic analysis identified a mutation in the endoglin gene, typical for patients with hereditary hemorrhagic telangiectasia (HHT), also called Osler–Weber–Rendu syndrome.

**Figure 5:**
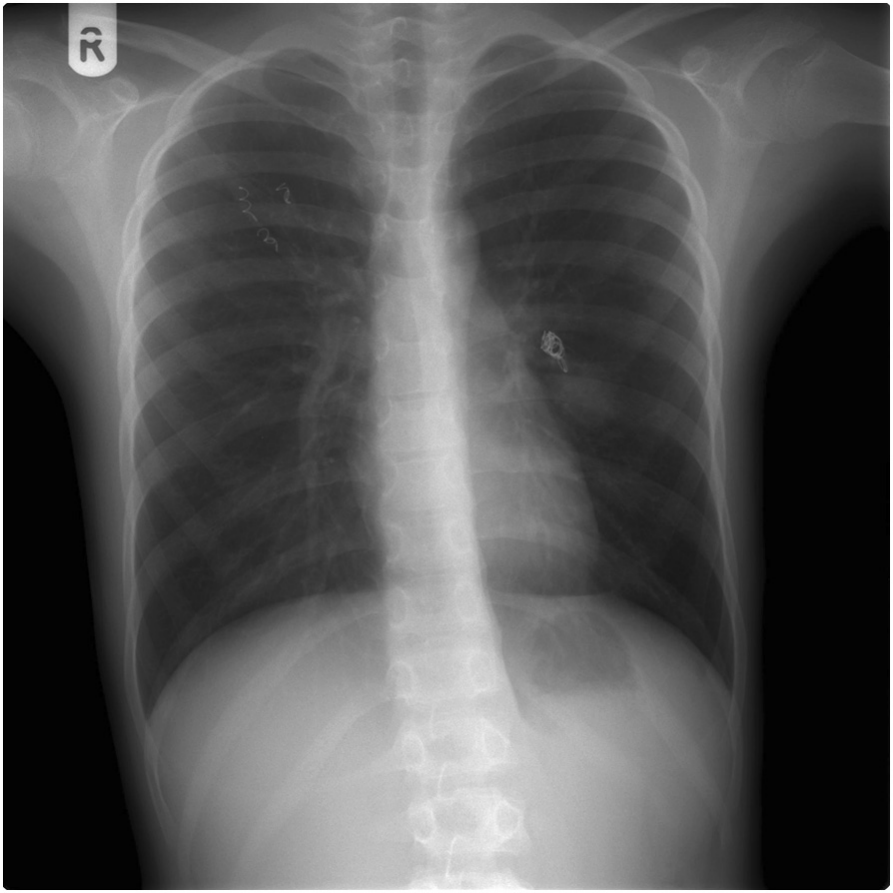
Chest X-ray with visible steels coils after embolotherapy of pulmonary arteriovenous malformations within the perihilar left upper lobe and small arteriovenous malformation within the apical segment of the right lobe.

**Figure 6:**
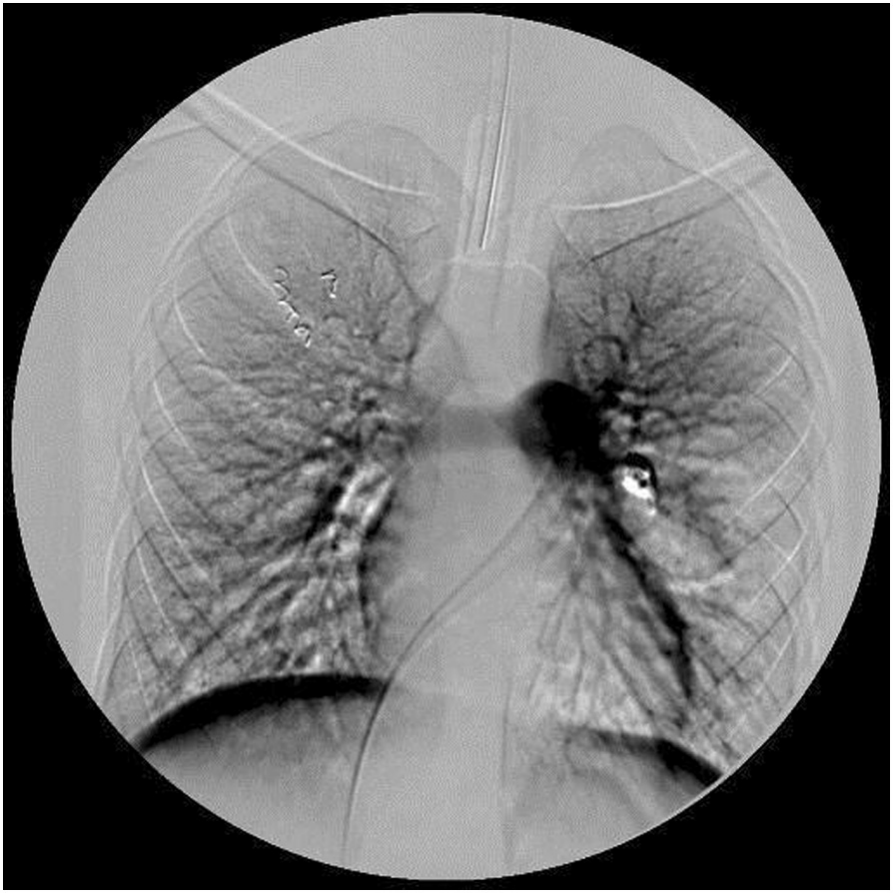
Angiogram following feeding artery embolization of both arteriovenous malformations.

Percutaneous transchateter embolization of both AVMs was performed with stainless steel coils (Figure 5). We used a transfemoral approach with selective catheterization of the feeding artery. We did not use an introducer catheter, and we only used a diagnostic catheter on the 0.035 hydrophilic guidewire. After we had achieved a secure position of the diagnostic catheter, we performed the embolization with stainless steel coils (Johnson-Johnson) whose official diameter was equal or 10% larger than the diameter of the feeding arteries. The control angiogram was performed with the same diagnostic catheter (Figures 3, 4). Post-embolotherapy, no residual flow was seen through the AVMs (Figure 6). The percutaneous pulse oxymetry saturation increased from 92% to 97% immediately on room air. His exercise tolerance improved and he was symptom free during the follow-up period of six months after the procedure. A magnetic resonance imaging (MRI) of the brain and cervical spine did not show any cerebral or cervical AVM. Further investigation revealed a positive family history: a cousin of the patient’s father has a diagnosed brain AVM.

## Discussion

The case of an 11-year-old Croatian boy of Mediterranean origin with a history of asthma, dyspnea on exertion and chronic hypoxemia is presented. The patient was initially misdiagnosed with difficult-to-control asthma, based on his past medical history, persistent symptoms of dyspnea, reduced exercise tolerance, and several asthma exacerbations. CT angiography of the thorax showed two PAVM, one on each side of the lungs. A three-dimensional reconstruction of the patient’s left lobe vascularization showed an AVM and an aneurism with a single feeding artery originating from the pulmonary artery, and a single draining vein. HHT clinical diagnosis was made by the presence of three Curaçao criteria (epistaxis, mucocutaneous telangiectases, and PAVM).

Many diseases mimic asthma and these alternate diagnoses should be considered, especially if asthma symptoms do not respond to treatment as expected [4]. According to the American Thoracic Society workshop consensus for definition of refractory or severe asthma certain criteria are used for diagnosis. First, major characteristics: a) treatment with continuous or near continuous (>50% of year) oral corticosteroids; and b) need for treatment with high-dose inhaled corticosteroids. Second, minor characteristics: a) one or more asthma exacerbations per year; b) use of a daily short-acting β2-agonist because of asthma symptoms; c) airway obstruction (FEV_1_ <80% of predicted, diurnal peak expiratory flow variability of >20%); d) needing three or more oral corticosteroid courses per annum; e) a near-fatal asthma event in the past; f) deterioration with reduction in oral or intravenous steroid dose; and g) needs additional daily treatment with a controller medication such as a long-acting β2-agonist, leukotriene antagonist, or theophylline [5]. Two major criteria, or one major criterion and two minor criteria must be met for diagnosis of difficult-to-control asthma [3]. In general, difficult-to-control asthma can be divided into two subgroups: first, true difficult-to-control asthma requiring thorough diagnostic work-up and appropriate management by a specialist; and second, false difficult-to-control asthma which is only apparently difficult-to-control and is conditioned by different comorbidities. Four different conditions can be classified under false difficult-to-control asthma. First, incorrect diagnosis of asthma: congenital anomalies of upper airways, large airways obstruction, gastro-esophageal reflux, cystic fibrosis, bronchiectasis, cardiac disease, and hyperventilation syndrome. Second, asthma and comorbid conditions. Third, continued exposure to aggravating factors. Fourth, non-adherence to treatment, incorrect choice of inhalers, and poor inhaler technique [3].

PAVM are uncommon. According to an autopsy study by Sloan and Cooley in 1953, only three cases of PAVM were detected in 15,000 consecutive autopsies [6]. The Mayo Clinic reported 194 cases of PAVM over 45 years, indicating an annual incidence of 4.3 cases per year [7-9]. PAVM may be acquired or congenital and approximately 70% of the cases of PAVM are associated with HHT [10]. PAVM occurs twice as often in the female gender compared with the male gender [11].

PAVM can be either simple or complex. The simple type (80% of cases) consists of a single feeding segmental artery and a single draining vein, and the complex type (20% of cases) has two or more feeding arteries or draining veins [12,13]. More than half of the lesions are in the lower lung fields, followed by the middle lobes and then the upper lobes [12,14]. PAVM can be single or multiple in occurrence and the incidence of single PAVMs ranges from 42% to 74% [8,9]. In patients with HHT the PAVM are multiple (35% to 65%) [8,15,16], and usually associated with severe complication and infection [17]. The study of Bosher *et al*. found that 25% of patients had bilateral disease [18]. Our patient had two simple PAVMs, one on each side of the lungs.

Approximately 72% of patients with PAVM have symptoms referable to PAVM or underlying HHT. Symptoms related to PAVM often develop between the fourth and sixth decades of life when the shunted blood exceeds 25% of total blood volume [6,19,20]. Symptoms of HHT commonly become noticeable before the age of 20 (for example, epistaxis, skin telangiectases) [21,22]. Dyspnea on exertion is the most common complaint in patients with PAVM and it is seen in about half of patients [15,23,24]. Other symptoms attributable to PAVM include the following: hemoptysis (in approximately 10% of patients), chest pain (6%), finger clubbing (20%), cyanosis (18%) and thoracic murmurs (3%) [15]. Our patient had dyspnea on exertion with chronic hypoxia and he was resistant to conventional asthma therapy. Thoracic murmur was revealed on physical examination.

Orthodeoxia is a decrease in partial pressure of oxygen in arterial blood (PaO_2_) or SaO_2_ that occurs when one assumes an upright position from the supine position. The fraction of cardiac output that shunts right-to-left circulation is elevated in patients with PAVM; normal values are less than 5%. The shunt fraction is most accurately assessed by using the 100% oxygen method, which involves the measurement of PaO_2_ and SaO_2_ after the patient breathes 100% oxygen for 15 to 20 minutes. A shunt fraction of more than 5% has a sensitivity of 87.5% and a specificity of 71.4% [10]. Spirometric findings are usually normal with mildly reduced diffusing capacity [15,25,26]. In our case, spirometry parameters were within the normal range and DLCO was decreased. The classic roentgenographic appearance of a PAVM is that of a round or oval mass of uniform density, frequently lobulated but sharply defined, more commonly in the lower lobes, and ranging from one to five cm in diameter. The sensitivity of chest radiograph alone is 70% in diagnosing PAVM [10]. In our patient, a chest X-ray showed a homogeneous soft tissue mass suspicious for tumor. CT angiography is considered the ‘gold standard test’ for the diagnosis of PAVM with sensitivity over 97% [27]. Our case also confirms this observation.

The natural course of PAVM is not benign. These lesions can be associated with a variety of life-threatening complications, such as stroke, brain abscess, hemothorax, and hemoptysis, especially in women [28,29]. Rupture of a PAVM can occur at any age, independently of lesion size [30-32]. Without appropriate treatment mortality exceeds 11% [33].

All symptomatic PAVMs and asymptomatic PAVMs larger than two cm, or if feeding arteries are larger than two mm, should be treated with surgery or embolotherapy because of the risk of paradoxical embolism [8,34]. The treatment of choice in patients with multiple or bilateral PAVM is transchateter embolotherapy with balloons or stainless steel coils, and vascular plugs (AMPLATZER®).

## Conclusion

PAVMs are relatively rare disorders with very common presentations. Detailed history taking and a high degree of suspicion are of immense importance for final diagnosis. Chronic hypoxemia in children with asthma requires additional diagnostic procedures, especially in cases not responding to oxygen supplemental therapy and conventional therapy.

## Consent

Written informed consent was obtained from the patient’s mother for publication of this manuscript and accompanying images. A copy of the written consent is available for review by the Editor-in-Chief of this journal.

## Abbreviations

AVM: Arteriovenous malformation; CT: Computed tomography; DLCO: Diffusing capacity of the lung for carbon monoxide; FEV_1_: Forced expiratory volume in one second; FVC: Forced vital capacity; HHT: Hereditary hemorrhagic telangiectasia; PaO_2_: Partial pressure of oxygen in arterial blood; PAVM: Pulmonary arteriovenous malformations; SaO_2_: Saturation level of oxygen in hemoglobin.

## Competing interests

The authors declare that they have no competing interests.

## Authors’ contributions

MN made substantial contributions to the analysis and interpretation of data, was involved in drafting the manuscript, and gave final approval of the version to be published. VV made substantial contributions to conception and design, revised the manuscript critically for important intellectual content, and gave final approval of the version to be published. FR made substantial contributions to acquisition of data, was involved in drafting the manuscript, and gave final approval of the version to be published. DE made substantial contributions to conception and design and acquisition of data, was involved in drafting the manuscript, and gave final approval of the version to be published. MT made substantial contributions to conception and design, revised the manuscript critically for important intellectual content, and gave final approval of the version to be published. All authors have read and approved the final manuscript.
